# Supplementary material for: Yellowing, Weathering and Degradation of Marine Pellets and Their Influence on the Adsorption of Chemical Pollutants
Source: Polymers (Basel). 2022 Mar 24;14(7):1305. doi: 10.3390/polym14071305 (PMC9003515; doi:10.3390/polym14071305)
Supplement: Supplementary file 1 [file polymers-14-01305-s001.zip › polymers-1596129-supplementary.pdf]

Table S1. Compounds list analized by GC-MS, classified as PCBs (group A), OCP (group B) or PAH (group C). Retention time (min) and SIM conditions for each compound.

SIM Groups	Analyzed Compounds				Retention Time (min)	Quantita-tion Ion	Qualifer Ion		Dwell Time (ms)
	Nº	Groups	Symbol groups	Compound Name					
1 (10.00-12.35min)	1	OCPs	B	AlphaBCH	11,465	219	181		50
	2	OCPs	B	Atrazine	11,911	215	217	220	50
	3	OCPS	B	BetaBCH	12,067	219	183		50
	4	OCPS	B	GammaBCH	12,242	219	181		50
2 (12.35-13.20min)	5	PAHs	C	Anthracene	12,63	178	176	179	60
	6	OCPs	B	DeltaBCH	12,815	219	181		60
3 (13.20-14.36min)	7	PCB	A	PCB28	13,68	258	186		75
	8	OCPs	B	Heptachlor	14,128	272	100		75
4 (14.36-16.87min)	9	PCBs	A	PCB52	14,635	292	220		75
	10	OCPs	B	Aldrin	15,113	263	293		75
5 (16.87-17.84min)	11	PCBs	A	PCB101	17,28	326	256		75
	12	OCPs	B	Endosulfan I	17,431	241	195		75
6 (17.84-18.62min)	13	OCPs	B	4,4-DDE	18,197	246	318		75
	14	OCPs	B	Dieldrin	18,254	263	79		75
7 (18.62-20.31min)	15	OCPs	B	Endrin	18,956	263	81		30
	16	PCBs	A	PCB118	19,289	326	254		30
	17	OCPs	B	4,4-DDD	19,564	235	165		30
	18	OCPs	B	Endrin Aldehyde	19,877	345	250		30
	19	PCBs	A	PCB153	20,024	360	290		30
8 (20.31-21.60min)	20	OCPs	B	Endosulfan Sulfate	20,661	272	387		50
	21	OCPs	B	4,4-DDT	20,794	235	165		50
	22	PCBs	A	PCB138	20,946	360	290		50
9 (21.60-24.50min)	23	PCBs	A	PCB180	23,184	396	324		150
10 (24.50-28.90min)	24	PCBs	A	PCB194	25,751	430	358		60
	25	PAHs	C	Benzo(b)fluoranthene	26,181	252	248	250	60
	26	PAHs	C	Benzo(a)pyrene	27,04	252	248	250	60
11 (28.90-32.00min)	27	PAHs	C	Indene(1,2,3-cd)-pyrene	29,997	276	275	274	100
